# Less functional variants of TLR-1/-6/-10 genes are associated with age

**DOI:** 10.1186/s12979-015-0034-z

**Published:** 2015-07-08

**Authors:** Lutz Hamann, Juozas Kupcinskas, Berrocal Almanza, Jurgita Skieceviciene, Andre Franke, Ute Nöthlings, Ralf R. Schumann

**Affiliations:** Institute of Microbiology and Hygiene, Charité University Medical Center Berlin, Charitéplatz 1, 10117 Berlin, Germany; Department of Gastroenterology, Lithuanian University of Health Sciences, Kaunas, Lithuania; Institute for Digestive Research, Lithuanian University of Health Sciences, Kaunas, Lithuania; University Hospital Schleswig Holstein, Campus Kiel, Schittenhelmstr. 12, Kiel, Germany; Popgen Biobank, Institute for Experimental Medicine, Christian-Albrechts-University Kiel, Niemansweg 11, Kiel, Germany; Present address: Department of Nutrition and Food Sciences, Rheinische Friedrich-Wilhelms-University Bonn, Endenicher Allee 11-13, 53115 Bonn, Germany

**Keywords:** Inflamm-aging, Innate immunity, Toll-like receptors, Polymorphisms, Healthy aging

## Abstract

**Background:**

Determining the prerequisites for healthy aging is a major task in the modern world characterized by a longer lifespan of the individuals. Besides lifestyle and environmental influences genetic factors are involved as shown by several genome-wide association studies. Older individuals are known to have an impaired immune response, a condition recently termed “inflamm-aging”. We hypothesize that the induction of this condition in the elderly is influenced by the sensitivity of the innate immune system. Therefore, we investigated genetic variants of the Toll-like receptor (TLR) family, one of the major family of innate immune receptors, for association with age in two cohorts of healthy, disease-free subjects.

**Results:**

According to sex we found a positive association of loss-of-function variants of TLR-1 and −6 with healthy aging with odds ratios of 1.54 in males for TLR-6 (249 S/S), and 1.41, 1.66, and 1.64 in females for TLR-1 prom., TLR-1 (248 S/S), and TLR-1 (602 S/S), respectively. Thus, the presence of these variants increases the probability of achieving healthy old age and indicates that a reduced TLR activity may be beneficial in the elderly.

**Conclusions:**

This is the first report showing an association of TLR variants with age. While a loss of function of an important immune receptor may be a risk factor for acute infections as has been shown previously, in the setting of healthy ageing it appears to be protective, which may relate to “inflamm-aging”. These first results should be reproduced in larger trials to confirm this hypothesis.

## Background

The immune system of the elderly is characterized by diminished effectiveness called immunosenescence. The mechanism for a decreased immune response in the elderly is complex and affects the innate as well as the adaptive immune system. Decreased diversity of the antigen repertoire resulting in diminished T- and B-cell responses, and accumulation of functionally impaired memory lymphocytes i.e. have been described to impair the adaptive immunity of the elderly [1]. Furthermore, a high-risk phenotype characterized by high CD8 and low CD4 cell numbers, has been suggested to be associated with higher mortality rate in a group of very old Swedish subjects [2].

However, functions of cells of the innate immune system may also be affected in older age: Key functions of neutrophils, macrophages, dendritic cells, and NK cells, e.g. chemotaxis, phagocytosis, migration, and cytotoxicity are known to be reduced in the elderly [3, 4]. One family of “Pattern recognition receptors (PRRs)” important for initiating the innate immune responses are the toll-like receptors (TLRs) recognizing “pathogen associated molecular patterns (PAMPs)” followed by the activation of the innate immune response [5]. Age-associated dysfunctions of human TLRs have been described before: An age dependent decrease in TLR-1/2 heterodimer function determined by a lessened TNF-α- and IL-6-release upon stimulation as well as an overall decrease in cellular TLR-1 expression have been described for monocytes of the elderly [6]. A much broader decrease in TLR function also including the intracellular RNA-sensors TLR-7 and −8 has been observed for dendritic cells (DCs) from aged subjects [7]. Single nucleotide polymorphisms (SNPs) leading to a loss of function of the respective TLRs therefore have been suspected to be associated with age. However, only a few studies focusing on TLR SNPs and aging are available today with conflicting results: A functional TLR-4 SNP has been shown to be overrepresented in centenarians and was suggested to be associated with a longer lifespan [8]. However this result could not be confirmed by a subsequent study [9].

Another important feature of immunosenescence is a chronic low-grade inflammation of the elderly, also termed “inflamm-aging”, characterized by increased plasma levels of inflammatory cytokines, acute phase proteins, and coagulation factors [10]. Most age-related diseases such as atherosclerosis, cardiovascular diseases, type 2 diabetes, and metabolic syndrome, representing the leading causes of death in the elderly of the Western world are characterized by chronic inflammation. Therefore, *inflamm-aging* could be seen as the common mechanism responsible for a general decline of health, and an onset of chronic inflammatory diseases in the elderly. The initiation of *inflamm-aging* is multifactorial and not completely understood today. However, it results in senescent cells exhibiting the so called “senescence associated secretory phenotype (SASP)” characterized by an increased release of pro-inflammatory cytokines [10]. One potential reason for *inflamm-aging* could be an increased “translocation” of bacteria from the gut as has been shown i.e. for HIV-infected subjects due to systemic immune activation, which is also an attribute of the elderly [11]. An increase of intestinal permeability with age has been shown in an animal model but could not be conclusively confirmed in humans [12, 13]. However, serum concentrations of lipopolysaccharide binding protein (LBP), a surrogate marker for microbial translocation, increase with age [14, 15]. Also the decline in adaptive immune functions could result in a higher bacterial load due to latent infections.

More recently it has been shown that an increased DNA damage response in the elderly also triggers the SASP phenotype [16]. Furthermore, free DNA or other cellular breakdown products recently also termed “danger-associated molecular patterns (DAMPS)” can also stimulate the release of pro-inflammatory cytokines by the innate immune system. Therefore, not only an increased bacterial load but also of cellular breakdown products increasing with age could affect the innate immune response and participate in the low-grade chronic inflammation of the elderly.

As described above various mechanisms are suspected to result in *inflamm-aging*, one of them might be a chronic inflammatory stimulus by PAMPs in response to microbial translocation or by DAMPs generated by cellular breakdown products. We hypothesize, that tolerance induction by these stimulatory compounds could take place in the elderly, which might explain in part the decreased inflammatory response of these individuals. If this is the case, loss of function SNPs within TLR genes decreasing the overall sensitivity of the innate immune system may increase the threshold level for tolerance induction and thereby protect from *inflamm-aging*. In addition, a decreased sensitivity of the innate immune system might also be beneficial in terms of chronic diseases. We have recently shown that loss of function genotypes of TLR-1 (I602S) and TLR-6 (P249S) are protective in chronic inflammatory disease such as gastric cancer and atherosclerosis [17, 18]. Although particularly for several types of cancer current data are conflicting [19] we speculate that these SNPs would be beneficial for healthy aging and should be associated with age in a healthy population. To test this hypothesis, we genotyped two cohorts of healthy volunteers, one from Germany and one from Lithuania, for 5 SNPs within the TLR1/6/10 cluster. We show here for the first time an age-dependent distribution for all of these SNPs indicating the less functional homozygote genotypes to be beneficial for healthy aging.

## Results

### The TLR-6/1/10 region and investigated SNPs

**Fig. 1:**
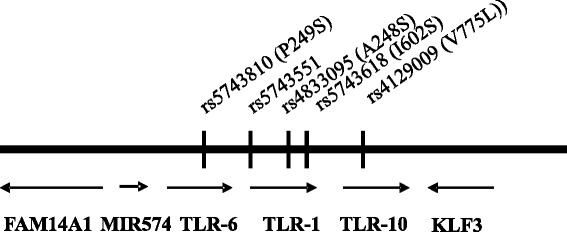
TLR-6/1/10 region on chromosome 4. Arrangement of TLR-6/1/10 and adjacent genes on chromosome 4 and the localization of investigated SNPs

Several SNPs within the TLR-6/1/10 genes located in close proximity to each other have been investigated in case/control studies before [20–25]. We focus here on 5 functionally relevant SNPs that have been extensively analyzed in vitro. Most detailed analyses were carried out for TLR-1 (I602S) indicating that the presence of this SNP inhibits the transport of the receptor to the cell surface resulting in a complete loss of function. TLR-1 (A248S) and TLR-6 (P249S) have been shown to reduce ligand-induced immune responses [26, 27]. The TLR-1 variant rs5743351 leads to a change of sequence within the promoter region and has been suggested to influence transcription leading to a change in cellular TLR-1 expression. The localizations of these SNPs and adjacent genes that may harbour SNPs potentially being in linkage disequilibrium are shown in Fig. 1.

### TLR-6/1/10 SNPs are associated with age

To analyze whether these SNPs are associated with age we genotyped two cohorts of healthy volunteers from Germany and Lithuania. Baseline characteristic of genotyped subjects are shown in Table 1 and more detailed information is presented in the Materials and Methods section. Results of genotyping for both cohorts indicate an unequal frequency distribution for TLR-1 and −6 SNPs in the German and for TLR-1 and −10 SNPs in the Lithuanian cohort with *P*-values ranging from 0.04 to <0.001 comparing young and old subjects (Table 2). The cut-off was set at age 50 because the onset of age-related diseases is minimal before the age of 50. Since sex is the most accepted factor associated with successful aging, with females reaching significantly higher age, we stratified our groups for sex. Functional studies for TLR-1 I602S and TLR-6 P249S suggested the homozygous mutated genotypes to be non or less functional regarding the initiated immune response, respectively. Therefore, we applied a recessive model to analyze association with age comparing homozygous mutated subjects with combined “wild-type” and heterozygous subjects. First we employed linear regression to show an association of the different genotypes with age over the whole range from age 25 to age 85.

**Table 1:** Basline characteristics of study subjects

	German cohort			Lithuanian cohort		
	All	Age < 50	Age ≥ 50	All	Age < 50	Age ≥ 50
	*N* = 1388	*N* = 865	*N* = 523	*N* = 1028	*N* = 772	*N* = 256
Mean age	43.3	30.09	65.43	42.6	37.4	58.4
SD	18.8	7.3	8.3	11.7	7.2	7.7
Age range		19–49	50–81		25–49	50–84
Male/female	768/620	394/471	374/149	631/397	516/256	115/141

**Table 2:** Genotype distribution in the German and Lithuanian cohort

German cohort	Young subjects (Age < 50, *n* = 866)			Old subjects (Age ≥ 50, *n* = 523)			*P*-value
	Wt (%)	Het (%)	Hom (%)	Wt (%)	Het (%)	Hom (%)	
TLR-6 (P249S)^a^	336 (38.8)	399 (46.1)	130 (24.9)	158 (30.2)	256 (48.9)	109 (20.8)	**0.001**
TLR-1	70	300	495	15	184	324	**<0.001**
prom.	(8.1)	(34.6)	(57.2)	(2.9)	(35.2)	(62.0)	
TLR-1	68	303	494	15	173	335	**<0.001**
(N248S)	(7.9)	(35.0)	(57.0)	(2.9)	(33.1)	(64.1)	
TLR-1	91	329	445	21	186	316	**<0.001**
(I602S)	(10.5)	(38.0)	(52.4)	(4.0)	(35.6)	(60.4)	
TLR-10	603	231	31	371	142	10	0.205
(V775L)	(69.6)	(26.7)	(3.6)	(70.9)	(27.2)	(1.9)	
Lithuanian cohort	Young subjects (Age < 50)			Old subjects (Age ≥ 50)			*P*-value
	Wt (%)	Het (%)	Hom (%)	Wt (%)	Het (%)	Hom (%)	
TLR-6 (P249S)	319	336	106	97	114	38	0.689
(*n =* 761, *n* = 250)	(41.9)	(44.2)	(13.9)	(38.8)	(45.6)	(15.2)	
TLR-1 prom.	25	222	515	4	52	194	**0.009**
(*n* = 762, *n* = 250)	(3.3)	(29.1)	(67.6)	(1.6)	(20.8)	(77.6)	
TLR-1 (N248S)	24	220	525	3	52	198	**0.008**
(*n* = 769, n = 251)	(3.1)	(28.6)	(68.3)	(1.2)	(20.7)	(78.9)	
TLR-1 (I602S)	29	242	498	7	58	189	**0.018**
(*n* = 762, *n* = 250	(3.8)	(31.8)	(65.4)	(2.8)	(23.2)	(75.6)	
TLR-10 (V775L)	575	166	7	209	38	1	**0.044**
(*n* = 748, *n* = 248)	(76.9)	(22.2)	(0.9)	(84.3)	(15.3)	(0.4)	

*P*-values were determined by 3×2 Chi^2^ comparing young and old subjects. Significant *P*-values below 0.05 were printed in bold. Different sample numbers in the Lithuanian cohort are due to the fact that some samples failed to be genotyped for all SNPs.^a^TLR-6 genotypes for this cohort have mostly been published previously [17]

Analyzing the association of less or non-functional homozygote genotypes with age by linear regression reveals significant associations for TLR-1 prom.: *P* = 0.039, TLR-1 (N248 S): *P* = 0.036, and TLR-1 (I602S): *P* = 0.014 in males and TLR-1 (I602S): *P* = 0.015 in females within the German cohort (Table 3). For the Lithuanian cohort we found significant associations only for females: TLR-1 prom.: *P* = 0.003, TLR-1 (N248S): *P* = 0.003, and TLR-1 (I602S): *P* = 0.032, and TLR-10 (V775L): *P* = 0.007 (Table 3). Combination of both groups yielded significant associations for TLR-6 in males and TLR-1 prom.,TLR-1 (N248S), TLR-1 (I602S) and TLR-10 (V775L) in females, *P* = 0.002, *P =* 0.005, *P =* 0.001, *P* = 0.032, and *P* = 0.032, respectively*.* Of note, significant associations for TLR-1 and 6 are accompanied with positive β-values. Significant associations of TLR-10 show negative β-values, indicating less functional genotypes for TLR-1 and −6 being overrepresented in the healthy elderly whereas the less functional TLR-10 genotype is underrepresented, which might be explained by the recent finding that TLR-10 in contrast to TLR-1 and 6 is an anti-inflammatory receptor [28]. However, the r^2^-values are rather low (0.001–0.021) indicating weak associations of these genotypes with age. We compared the correlation between all TLR-1 variants and found the correlation coefficients to be >0.875, *P* = 0.001 (data not shown) suggesting that the three TLR-1 SNPs are in strong linkage disequilibrium.

**Table 3:** Associations of less functional TLR-1/6/10 genotypes with age

Predictor German cohort	Male					Female				
	β	S.E	R^2^	P	95 % CI	β	S.E	R^2^	P	95 % CI
TLR6 (249S/S)	−0.69	0.75	0.001	0.122	−2.17–0.78	−1.20	0.77	0.002	0.355	−2.71–0.32
TLR-1 prom	2.24	1.13	0.004	**0.039**	0.12–4.56	0.81	1.12	0.001	0.470	−1.39–3.01
TLR-1 (248 S/S)	2.38	1.13	0.004	**0.036**	0.80–4.05	2.02	1.13	0.004	0.074	0.80–4.05
TLR-1 (602 S/S)	2.68	1.05	0.007	**0.014**	0.55–4.82	2.57	1.09	0.008	**0.015**	0.51–4.63
TLR-10 (775 V/V)	−0.39	1.28	0.001	0.760	−2.89–2.11	−0.39	1.33	0.001	0.770	−3.00–2.22
Lithuanian cohort										
TLR6 (249S/S)	0.97	0.61	0.002	0.112	−0.23–2.16	−0.97	0.91	0.001	0.288	−2.77–0.83
TLR-1 prom	1.11	0.82	0.001	0.178	−0.51–2.72	3.41	1.15	0.020	**0.003**	1.15–5.67
TLR-1 (248 S/S)	1.08	0.82	0.001	0.189	−0.53–2.66	3.56	1.17	0.021	**0.003**	1.26–5.86
TLR-1 (602 S/S)	1.33	0.79	0.003	0.093	−0.22–2.87	2.36	1.10	0.009	**0.032**	0.20–4.51
TLR-10 (775 V/V)	−1.10	0.96	0.001	0.254	−2.99–0.79	−4.13	1.53	0.016	**0.007**	−7.12–1.13
Combined										
TLR6 (249S/S)	1.88	0.61	0.006	**0.002**	0.70–3.07	0.74	0.74	0.001	0.310	−0.70–2.19
TLR-1 prom	1.05	0.76	0.001	0.170	−0.45–2.54	2.40	0.85	0.007	**0.005**	0.74–4.06
TLR-1(248 S/S	1.03	0.76	0.001	0.176	−0.46–2.52	3.24	0.85	0.013	**0.001**	1.56–4.91
TLR-1 (602 S/S)	1.21	0.73	0.001	0.096	−0.22–2.64	3.24	0.80	0.015	**0.032**	1.68–4.80
TLR-10 (775 V/V)	0.21	0.87	0.001	0.813	−1.50–1.19	−2.25	1.05	0.004	**0.032**	−4.30–0.20

Analysis was carried out by univariate linear regression. Significant *P*-values below 0.05 were printed in bold

### Homozygous loss-of-function genotypes are more frequent in the Elderly

The rather low r^2^-values obtained by linear regression analysis may be explained by the mechanisms of action of these SNPs. Given that these SNPs may influence the onset of common diseases associated with age or the development of *inflamm-aging* we speculate that an association with age would be more visible comparing older subjects with younger subjects, than analyzing age association over the whole range by linear regression analysis. Since the onset of age-related diseases is around the age of 50, we divided both cohorts in young subjects (age < 50 years) and old subjects (age ≥ 50 years) and assessed the probability of achieving the old subjects group as outcome variable in dependence of the genotype-distribution as predictor variable by univariate logistic regression analysis (Table 4). Univariate analysis yielded significant associations for TLR-1 (248S/S) and TLR-1 (602S/S) in the German cohort for males with the following Odds ratios (OR): 1.36 (95 % CI: 1.02–1.81) *P*-value: *P* = 0.038 and OR: 1.41 (95 % CI: 1.06–1.87), *P* = 0.02, respectively. For females we found only TLR-1 (602S/S) to be significantly associated, OR: 1.53 (95 % CI: 1.05–2.23) *P* = 0.027, (Table 4). Significant associations for the Lithuanian cohort were as follows: Males: TLR-1 (602S/S), OR: 1.77 (95 % CI: 1.09–2.86), *P*-value: 0.02 and for females: TLR-1 prom, TLR-1 (248S/S), TLR-1 (602S/S) and TLR-10 (775 V/V) with OR: 1.44 (95 % CI: 1.13–1.83); *P* = 0.003, OR: 2.12 (95 % CI: 1.30–3.43), *P* = 0.002, OR: 1.62 (95 % CI:1.04–2.53); *P* = 0.033 and OR: 0.52 (0.31–0.89), *P* = 0.016, respectively.

**Table 4:** Homozygous less functional genotypes are increased in older subjects

	Male		Female	
German	Odds ratio (95 % CI)	*P*-value	Odds ratio (95 % CI)	*P*-value
TLR-6 (249S/S)	1.41 (0.98–2.04)	0.067	1.56 (0.97–2.50)	0.066
TLR-1 prom.	1.31 (0.98–1.76)	0.065	1.02 (0.70–1.48)	0.93
TLR-1 (248S/S)	1.36 (1.02–1.81)	**0.038**	1.31 (0.90-1.92)	0.17
TLR-1 (602S/S)	1.41 (1.06–1.87)	**0.02**	1.53 (1.05–2.23)	**0.027**
TLR-10 (775 V/V)	1.05 (0.77–1.44)	0.741	1.01 (0.69–1.54)	0.89
Lithuanian				
TLR-6 (249S/S)	1.53 (0.89–2.62)	0.123	0.88 (0.65–1.89)	0.394
TLR-1 prom.	1.46 (0.91–2.36)	0.117	1.44 (1.13–1.83)	**0.003**
TLR-1 (248S/S)	1.42 (0.88–2.83)	0.152	2.12 (1.30–3.43)	**0.002**
TLR-1 (602S/S)	1.77 (1.09–2.86)	**0.02**	1.62 (1.04–2.53)	**0.033**
TLR-10 (775 V/V)	0.13 (0.36–1.14)	0.132	0.52 (0.31–0.89)	**0.016**
Combined				
TLR-6 (249S/S)	1.54 (1.15–2.05)	**0.003**	1.16 (0.79–1.68)	0.44
TLR-1 prom.	1.12 (0.89–1.41)	0.34	1.41 (1.06–1.89)	**0.018**
TLR-1 (248S/S)	1.13 (0.89–1.42)	0.32	1.66 (1.24–2.23)	**0.001**
TLR-1 (602S/S)	1.18 (0.94–1.48)	0.15	1.64 (1.28–2.19)	**0.001**
TLR-10 (775 V/V)	0.97 (0.75–1.25)	0.79	0.73 (0.54–1.01)	0.05

Analysis was carried out by univariate logistic regression comparing the less functional homozygote genotype with the others in young versus old subjects. The reference were young subjects and the wild type + heterozygous genotype. Significant *P*-values below 0.05 were printed in bold

The combination of both cohorts yielded significant associations for TLR-6 in males and for all TLR-1 and TLR-10 variants in females (Table 4); for TLR-6 (249S/S) in males OR: 1.54 (95 % CI: 1.15–2.05) *P*-value 0.003, in females for TLR-1 prom., 248S/S, and 602S/S OR: 1.41 (95 % CI: 1.06–1.89) *P* = 0.018, OR: 1.66 (95 % CI: 1.24–2.23) *P* = 0.001 and OR 1.64 (95 % CI: 1.28–2.19) *P* = 0.001, respectively. The TLR-10 variant 775 V/V showed a borderline significant association OR 0.73 (95 % CI: 0.54–1.01) *P* = 0.05. Again, as shown by linear regression analysis significant associations for TLR-10 are accompanied by OR’s below 1, whereas significant associations for TLR-1 and 6 are accompanied by OR’s over 1. For the combined analysis we performed a power analysis showing the power to be >82 % for all significant associations with the exception of the borderline association of TLR-10 with a power of 50 %.

In order to determine which age group shows the strongest increase of the homozygous less functional genotypes we compared the genotype distribution in young subjects (19–45 years, male: *n* = 910, female: *n* = 727) with subjects of 50–64 years (male: *n* = 281, female: *n* = 176), 65–74 years (male: *n* = 148, female: *n* = 70), and 75–85 years (male: *n* = 60, female: *n* = 40), respectively. This analysis was employed for the combined cohort only since separate analyses of the two cohorts would lead to low sample numbers of the subgroups. As shown in Table 5 comparison of young subjects with subjects of 50–64 years yielded nearly the same significant associations with comparable *P*-values and slightly enhanced ORs. Comparison of the older subgroups gave no significant associations most likely due to low sample numbers in these groups. However, the decreased OR’s in these groups compared to the group of 50–64 years may indicate that these associations are not present in older subjects.

**Table 5:** Homozygous less functional genotypes are especially increased in 50–64 years old subjects

Combined cohort	Male		Female	
TLR-6 (249 S/S)	Odds ratio (95 % CI)	*P*-value	Odds ratio (95 % CI)	*P*-value
50–64 years	1.52 (1.07–2.15)	**0.019**	1.51 (0.99–2.30)	0.056
65–74 years	1.47 (0.94–2.29)	0.093	0.55 (0.23–1.29)	0.170
75–85 years	1.83 (0.98–3.43)	0.059	0.91 (0.37–2.20)	0.827
TLR-1 prom.				
50–64 years	1.17 (0.88–1.56)	0.271	1.79 (1.24–2.57)	**0.002**
65–74 years	1.03 (0.71–1.48)	0.889	1.11 (0.66–1.85)	0.697
75–85 years	1.11 (0.64–1.93)	0.707	0.90 (0.49–1.67)	0.742
TLR-1 (248S/S)				
50–64 years	1.22 (0.91–1.62)	0.180	1.85 (1.28–2.66)	**0.001**
65–74 years	1.01 (0.70–1.46)	0.946	1.49 (0.87–2.54)	0.149
75–85 years	1.02 (0.59–1.76)	0.947	1.31 (0.69–2.49)	0.407
TLR-1 (602S/S)				
50–64 years	1.33 (1.00–1.76)	**0.047**	1.76 (1.25–2.50)	**0.001**
65–74 years	1.09 (0.76–1.56)	0.647	1.50 (0.90–2.49)	0.122
75–85 years	0.87 (0.51–1.47)	0.591	1.45 (0.77–2.73)	0.245
TLR-10 (775 V/V)				
50–64 years	0.99 (0.73–1.36)	0.971	0.69 (0.46–1.01)	0.057
65–74 years	1.12 (0.76–1.669	0.569	0.65 (0.36–1.17)	0.149
75–85 years	1.01 (0.55–1.85)	0.98	1.11 (0.58–2.13)	0.763

Analysis was carried out by univariate logistic regression comparing the less functional homozygote genotype with the others in different age groups. The reference were 19–49 years old subjects (male: *n* = 910, female: *n* = 727) and the wild type + heterozygous genotype. Significant *P*-values below 0.05 were printed in bold

## Discussion

We show here for the first time an association of SNPs within the TLR-6/1/10 cluster and age. The less functional genotypes of TLR-1 and −6 are positively associated with age as indicated by positive β-values (linear regression, Table 3) or OR’s above 1 (logistic regression, Table 4). In contrast, the mutated TLR-10 genotype shows a negative association with age as indicated by negative β values or OR’s below 1. This could be explained by the recent finding that TLR-10, in contrast to TLR-1 and 6, is viewed as an anti-inflammatory receptor [28]. Although the results differ slightly between the two study groups potentially due to ethnic variations, we conclude that these genotypes may be beneficial in terms of healthy aging. Analyzing the combined cohort for different age groups showed that these results are most prominent in subjects of 50–64 years compare to young subjects of 19–49 years (logistic regression, Table 5).

Differences in life span have been attributed to various factors including lifestyle, environmental factors, sex, and genetics, which may account for life span determination of about 30 % [29]. Several genome-wide association studies have been carried out to identify polymorphisms associated with longevity. Associations have been found that were confirmed by several studies, e.g. variants within the CETP-, APOC-3-, and ADIPOQ-genes [30, 31]. An association of the TLR-6/1/10 region has never been reported by these GWAS. However, in this study we aimed to investigate the genetics of a healthy life-span, which is clearly different from longevity. It has been shown that lack of risk alleles for age-associated common diseases are not necessarily associated with longevity [32, 33]. We therefore compared a healthy middle-aged control group younger than 50 years with a healthy group of aged subjects with an age ranging from 50 to 80 years. We set the cut-off at age 50 because it is assumed that in modern developed countries mortality due to age-related diseases before the age of 50 is minimal. Therefore, in contrast to our older group of healthy subjects the control group is a nearly unselected group regarding age-related morbidity.

Age of 50+ years is characterized by the onset of common age-associated diseases and *inflamm-aging* [34]. In line, the increase of serum levels of LBP, an acute-phase marker starts at an age of 50 in healthy subjects, which may indicate a change within the innate immune response [14]. It has been speculated that a sensitive immune system, which is clearly beneficial during young age may be harmful for the elderly [34]. This point of view fits well to the mechanism of antagonistic pleiotropy suggested to be involved in age-dependent diseases and longevity [30]. Our finding of less functional TLR-6/1/10 genotypes being over-represented in the healthy elderly further supports this view of aging. An earlier study failed to find an association of two TLR-1 SNPs (N248S, I602S) with age, however, in this study nonagenarians were compared with old controls (66–89 years) [35], which is a different approach as compared to the one used here. Our study suggests that the beneficial effects of the less functional genotypes is most prominent at the age of 50–64 years. This period of life span is the time of onset of age related diseases which are in part characterized by chronic inflammation, indicating that these variants maybe involved in this inflammatory process.

The mutated allele of an intergenic SNP between TLR-1 and −6 has been shown to be associated with allergic sensitization [36]. This SNP exhibits a similar distribution in Africa and Europe as found for the TLR-1 SNPs we investigated suggesting a strong linkage. In line, an earlier study investigating several SNPs within TLR-1 and −6 (P249S) showed that they are associated with an increased risk for childhood asthma [37]. These results, taken together with the results presented here, also fit to the theory of antagonistic pleiotropy.

A mechanism explaining this could be the development of immune-tolerance in the elderly by increased immune stimuli in aged people by microbial translocation or cellular breakdown products [38]. Tolerance has been described first as endotoxin (LPS)-induced tolerance where exposure of immune cells with low concentrations of LPS results in a refractory state towards a secondary stimulation [39]. More recently it has been shown that cross-tolerance also could be induced by stimulation with low concentrations of TLR2/1 agonists resulting in tolerance against a second stimulation with LPS [40]. Development of immune-tolerance depends on the detection of minute amounts of bacterial or danger associated stimuli. Variants that affect the sensitivity of the innate immune system should influence this process. Furthermore, variants that lower the sensitivity of the innate immune system could also be beneficial in terms of age associated chronic inflammatory diseases. However, this has to be investigated and proven in further studies.

Another interesting feature of TLR6/1/10 SNPs is the high variation in allelic frequencies observed among different ethnia: In African populations the TLR-1 ”wt”-alleles account for nearly 90 %, whereas in Europe the “mutated” allele has a frequency of around 75 %. This difference could be connected to antagonistic pleiotrophy since evolutionary pressure should be quite different due to different infectious diseases, lifestyle and environmental factors within the different continents. Other explanations for beneficial effects of less functional TLR6/1/10 genotypes might be a possible linkage with SNPs within genes nearby, for example Mir-574 located adjacent to TLR-6. Mir-574 has been associated with outcome of sepsis and may therefore be an important regulator of the immune responses [41]. Of note, it has been shown recently that TLR-1 and −6 less functional genotypes are associated with an enhanced immune response after BCG vaccination [42] potentially indicating a completely different mechanism by which these SNPs could be involved in healthy aging.

Since our conclusions are speculative we will discuss the limitations of our study in detail here: The advantage of our study is that we have two ethnically different populations with a sample size yielding sufficient statistical power showing similar results. The limitation due to the retrospective character of our study is the insufficient characterization of the study subjects regarding confounding risk factors. Although all participants reported to not have had a chronic disease or regularly use of medication at recruitment time, in future studies these important points may have to be controlled more thoroughly and additional confounding risk factors e.g. smoking, alcohol consume and others have to be checked carefully in order to confirm our findings. However, in light of the current research on genetic determinants of human health span we think that our results present an important first link of the involvement of TLR-6/1/10 polymorphisms in healthy ageing.

## Conclusion

We present here first evidence for a positive association of less functional TLR variants with increased age in healthy subjects. This association is most prominent in subjects of 50–64 years, suggesting these variants to be involved in the onset of age related diseases. However, this hypothesis needs to be confirmed by further studies.

## Methods

### Study subjects

#### German cohort

DNA from 900 healthy controls obtained from the *PopGen* biobank (University of Kiel, Germany) and from further 488 healthy controls collected at the Institute of Microbiology, Charité University Medical Center Berlin were included for the German cohort. Participants reported to not have had a chronic disease or regularly use medication at recruitment. All participants were of Caucasian ethnicity, the samples obtained from *PopGen* biobank were from residents of Schleswig Holstein, the northern part of Germany. *PopGen* biobank represents a population-based cohort. This cohort has been described previously showing an age-dependent distribution of the TLR-6 P249S genotype [17]. Slightly different sample numbers are due to the inclusion of some additional subjects and the exclusion of some samples that could not be analyzed for all SNPs under investigation. The study procedures followed were in accordance with the Helsinki Declaration of 1975, as revised in 1983 and was approved by the Ethical Board of the Charité - Universitätsmedizin Berlin and University of Kiel, respectively, and written informed consent was obtained from all participants. This study were approved by the local ethics committees.

#### Lithuanian cohort

Blood of 1028 healthy voluntary unrelated blood donors, nearly representing the typical Lithuanian overall population, was obtained during the years 2008–2012 at National Blood Center (Kaunas, Lithuania). All participants were of Caucasian ethnicity and were free of chronic disease or permanent medication use. The study was approved by Lithuanian Bioethics committee (Protocol No. 2/2008) and all subjects have signed an informed consent form to participate in the study. DNA from whole blood was extracted using salting-out method. This cohort has been previously used for hemochromatosis gene *HFE* studies [43]. All participants were of Caucasian origin and both parents were born in Lithuania. This study were approved by the local ethics committees, and carried out in accordance with the ethical guidelines of the 1975 Declaration of Helsinki.

### Genotyping

Genomic DNA was prepared by standard procedures from whole blood. Genotyping was carried out by PCR including fluorescence-labeled hybridization FRET probes followed by melting curve analysis employing the LightCyler 480™ (Roche Diagnostics). Genotyping for TLR-6 SNP P249S (rs5743810), TLR-1 A248S (rs4833095), TLR-1 I602S (rs5743618) was described previously [17, 44, 45]. Primer and probes were as follows: rs5743810: f-primer: gaaagactctgaccaggcat, r-primer: ctagtttattcgctatccaagtg, anchor probe: LC640-ttaccctcaaccacat agaaacgacttgga, sensor probe: accagaggtccaaccttactgaa-FL; rs4833095: f-primer: ttggatgtgtca gtcaagactgtag, r-primer: gcttcacgtttgaaattgag, anchor probe: LC640-gtttgaagtttcgccagaatacttag g, sensor probe: ttaaggtaagacttgataactttgg-FL; rs5743618: f-primer: tgtgactacccggaaagttataga, r-primer: cccagaaagaatcgtgcc, anchor probe: LC640-cctccctctgcatctacttggat, sensor probe: cca tgctggtgttggctgtgactgtg-FL.

TLR-1 prom (rs5743551): Melting curve analysis was carried out employing following primers: f-primer: gcatatttttactgccctgaatc and r-primer: tggctcccaaaggcattgac, anchor probe: LC640-actgccctgcccacttccct, and sensor probe: ttcagagtgctgagaagcttccc-FL resulting in melting points of 63 °C and 57 °C for the wild-type, and the mutated allele, respectively.

TLR-10 V775L (rs4129009): Melting curve analysis was carried out employing following primers: f-primer: gagactcttcatttaactctgtga and r-primer: cccaaggataggcgtaa , anchor probe: LC640-agccaccagagaaatgtatgaactgca, and sensor probe: cttcgagctgctatta-atgttaatgtat-FL resulting in melting points of 60 °C and 56 °C for the wild-type, and the mutated allele, respectively. Primers and probes were designed by O. Landt (TIB-MOLBIOL, Berlin, Germany).

### Statistics

Linear and logistic regression analyses have been performed by employing the IBM SPSS Statistics software package (version 20.0, IBM, Munich, Germany). Power analysis was carried out online by the openepi webside (http://www.openepi.com).
