# Erratum: Less functional variants of TLR-1/-6/-10 genes are associated with age

**DOI:** 10.1186/s12979-015-0037-9

**Published:** 2015-08-11

**Authors:** Lutz Hamann, Juozas Kupcinskas, Luis C. Berrocal Almanza, Jurgita Skieceviciene, Andre Franke, Ute Nöthlings, Ralf R. Schumann

**Affiliations:** Institute of Microbiology and Hygiene, Charité University Medical Center Berlin, Charitéplatz 1, Berlin, 10117 Germany; Department of Gastroenterology, Lithuanian University of Health Sciences, Kaunas, Lithuania; Institute for Digestive Research, Lithuanian University of Health Sciences, Kaunas, Lithuania; University Hospital Schleswig Holstein, Campus Kiel, Schittenhelmstr. 12, Kiel, Germany; Popgen Biobank, Institute for Experimental Medicine, Christian-Albrechts-University Kiel, Niemansweg 11, Kiel, Germany; Present address: Department of Nutrition and Food Sciences, Rheinische Friedrich-Wilhelms-University Bonn, Endenicher Allee 11-13, Bonn, 53115 Germany

After publication of this study [1], the authors noticed that the spelling of author Luis C Berrocal Almanza’s name was incorrect. The original version of this article has been updated to correct this. The publisher apologises for any inconvenience caused.
